# The Danish Spine Surgical Society is affiliated with brain and Spine: Together for the future of spinal surgery

**DOI:** 10.1016/j.bas.2025.104315

**Published:** 2025-07-03

**Authors:** Mikkel Mylius Rasmussen, Mikkel Østerheden Andersen

**Affiliations:** Danish Spine Surgical Society, Denmark

The Danish Spine Surgical Society is a non-specialty bearing society within the Organization of Dansih Medical Scientific Societies, which serves as an umbrella organization for medical scientific societies in Denmark. The Danish Spine Surgical Society aims to advance spinal surgical medical science, strengthen the quality of surgical spinal care in Denmark, and influence national health policy. All of this with the overall aim to improve outcomes for Danish patients undergoing spinal surgery. The board of The Danish Spine Surgical Society is composed so that there are always two specialists from the Danish Orthopedic Society and two specialists from Danish Neurosurgical Society serving as board members.

The Danish Spine Surgical Society aim to promote scientific and clinical work within the spinal field, including:

- Organizing scientific meetings, including at least one meeting as a scientific and professional annual meeting.
- Advancing surgical education within the spinal field.
- Fostering understanding and collaboration between spinal surgical specialties in Denmark.
- Expanding national and international relations for surgical spinal treatment, both in research and in clinical practice.
- Acting as an advisory function to third parties.

The society was established in 1999 and has experienced great support ever since. It is the primary forum for spinal surgeons in Denmark. Currently, there are 113 members of the society; approximately 40 % are neurosurgeons and 60 % are orthopedic surgeons. Membership is open to physicians who are members of either the Danish Orthopedic Society or Danish Neurosurgical Society and have an interest in spinal surgery. Additionally, other doctors with documented interest in spinal surgical diagnosis and treatment can join as members. Foreign doctors or individuals who have shown special interest in Danish spinal surgery may be accepted as corresponding members, and honorary members may be admitted under special circumstances.

The Danish Spine Surgical Society aims to engage in international networks and strengthen organizational and scientific collaborations. In addition to its affiliations with the Danish Orthopedic Society and the Danish Neurosurgical Society, and other medical scientific societies in Denmark, the society is part of the European Network for National Spine Societies (EUSSAB) within the EuroSpine family. Currently, The Danish Spine Surgical Society is working to formalize spinal surgical education in Denmark and has established a network to support national or multi-institutional scientific projects.

One of Societies' key focuses is managing DaneSpine—a national spinal surgical database that collects surgery data and Patient Reported Outcome Measures (PROM) from patients who have undergone spinal procedures. The database was launched in 2009 and currently contains more than 116,000 patients. It is based on voluntary collaboration between the country's spinal surgical departments and clinics. A national secretariat supports DaneSpine’s daily operations. Currently, data are collected from 17 different clinics across the country—7 private clinics and 10 public clinics. The ambition is to create a nationwide database that systematically collects data about spinal surgical patients, operative procedures, and outcomes. This allows for quality assessment and, if necessary, improvement of procedures and treatments for patients who have undergone spinal procedures in Denmark. The database is built with modules that enable comparison with data from similar databases in Sweden (SweSpine) and Norway (NorSpine). In this way The Danish Spine Surgical Society supports quality monitoring and scientific work in the Danish spinal surgery institutions.

As part of a desire to further strengthen our scientific engagement, The Danish Spine Surgical Society decided at the society's annual meeting in May 2025 to apply for affiliation with Brain and Spine. We are very pleased with the initiative to support national spinal surgical societies' scientific activities, and we are delighted to have received acceptance from Brain and Spine. Brain and Spine is already the official journal for the European organizations EuroSpine and the European Association of Neurosurgical Societies (EANS), and with its profile as an open-access platform dedicated to supporting spinal surgical research broadly, it is an excellent match for The Danish Spine Surgical Societyas a scientific society founded on classic European democratic values.

We look forward to the collaboration and to support the continued positive development of Brain and Spine. Among the many opportunities it offers us as a society is to publish the award-winning paper from our annual scientific meeting. We will be delighted to take advantage of this opportunity starting in 2026.

